# Significant improvement in quality of life following surgery for hydrocoele caused by lymphatic filariasis in Malawi: A prospective cohort study

**DOI:** 10.1371/journal.pntd.0008314

**Published:** 2020-05-08

**Authors:** Hannah Betts, Sarah Martindale, John Chiphwanya, Square Z. Mkwanda, Dorothy E. Matipula, Paul Ndhlovu, Charles Mackenzie, Mark J. Taylor, Louise A. Kelly-Hope

**Affiliations:** 1 Centre for Neglected Tropical Diseases, Department of Tropical Disease Biology, Liverpool School of Tropical Medicine, Liverpool, United Kingdom; 2 Ministry of Health, Lilongwe, Malawi; University of Colorado, UNITED STATES

## Abstract

**Background:**

Lymphatic filariasis (LF) is a mosquito-borne parasitic infection that causes significant disabling and disfiguring clinical manifestations. Hydrocoele (scrotal swelling) is the most common clinical condition, which affects an estimated 25 million men globally. The recommended strategy is surgical intervention, yet little is known about the impact of hydrocoele on men’s lives, and how it may change if they have access to surgery.

**Methodology/Principal findings:**

We prospectively recruited and followed-up men who underwent surgery for hydrocoele at six hospitals in an LF endemic area of Malawi in December 2015. Men were interviewed at hospitals pre-surgery and followed-up at 3-months and 6-months post-surgery. Data on demographic characteristics, clinical condition, barriers to surgery, post-surgery symptoms/complications and quality of life indicators were collected and analysed pre- and post-surgery, by age group and stage of disease (mild/moderate vs. severe), using chi-square tests and student’s t test (paired).

201 men were interviewed pre-surgery, 152 at 3-months and 137 at 6-months post-surgery. Most men had unilateral hydrocoeles (65.2%), mild/moderate stages (57.7%) with an average duration of 11.4 years. The most reported cause of hydrocoele was it being sexually transmitted (22.4%), and the main barrier to surgery was the cost (36.3%). Pre-surgery, a significant difference in the scrotum side affected was found by age group (X^2^ = 5.978, p = 0.05), and men with severe stage hydrocoele reported more problems with their quality of life than those with mild/moderate stage (t = 2.793; p = 0.0006). Post-surgery, around half of the men reported some pain/discomfort (55.9%), swelling (8.6%), bleeding (3.3%) and infection (5.9%), most of which had resolved at 3-months when the most significant improvements in their quality of life were found (t = 21.3902; p = 0.000). Post-surgery at 6 months all men reported no physical, social, psychological problems and took no time off work.

**Conclusion/Significance:**

Surgery had a significant positive impact on many aspects of a patient’s life, and the expansion of this treatment to all those affected in LF endemic areas would greatly improve the quality of men’s and their families’ lives, and greatly contribute to the global goal of providing universal health care.

## Author summary

Lymphatic filariasis is a mosquito-borne infection that causes significant disabling and disfiguring clinical conditions. Hydrocoele (scrotal swelling) is the most common condition affecting an estimated 25 million men worldwide. Surgery is the main intervention, however, there is limited information on its impact. This study assessed men before and after surgery in an endemic region of Malawi, to identify potential barriers and determine how surgery may change the quality of their life. Before surgery, 201 men reported to have moderate to severe problems with many aspects of their life including pain, mobility, usual activities, self-care, social issues, psychological health and ability to work. The main barrier to hydrocoele surgery was the cost, including associated transport and accommodation costs. After surgery, follow-up surveys at 3-months and 6- months found that men reported minimal or no physical, social, psychological problems and took no time off work. Surgery had a significant positive and lasting impact on men’s lives. National programmes need to advocate for resources to increase funding to support men and the uptake and demand for quality surgical services.

## Introduction

Lymphatic filariasis (LF) is a mosquito-borne parasitic infection of significant public health importance in 73 countries worldwide [1]. Filarial infection impairs the function of lymphatic vessels and associated tissues and can lead to chronic disabling and disfiguring conditions including hydrocoele (scrotal swelling), lymphoedema (tissue swelling) and elephantiasis (skin/tissue thickening) of limbs; these are most often accompanied by acute inflammation or acute dermato-lymphangio-adenitis (ADLA). LF is a neglected tropical disease (NTD) and one of the leading causes of permanent and long-term disability[2], with an estimated 65 million people affected globally. LF accounts for at least 2.8 million disability-adjusted life years (DALYs), but recent studies estimate the DALY burden to be nearly double this when taking different measures into account [3–5]. The Global Programme to Eliminate LF (GPELF) aims to address this burden and alleviate suffering through the management of morbidity and disability prevention (MMDP)[1], and advocates that national LF elimination programmes implement a minimum package of care, including surgery for hydrocoele and management of lymphoedema to prevent progression of disease and ADLAs.

Hydrocoele is the most common LF clinical condition with an estimated 25 million men affected globally, yet has not received adequate attention despite being able to be treated through surgery [2,6–10]. The accumulation of fluid in the tunica vaginalis causes the enlargement of the scrotum, and resulting effects on the penis, can collectively impact men’s physical and sexual function, marriageability, social life, psychological health, work and earning capacity, leaving them subject to social stigmatisation, ridicule, shame and poverty [4,11–19]. National MMDP programmes need to scale-up hydrocoele surgeries and improve capacity by training and integrating cases into routine hospital care, or by conducting hydrocoele campaigns (often referred to as hydrocoele surgical ‘camps’) where mass surgery is conducted over a few days to a week in a local facility.

Hydrocoelectomy is one of 28 essential surgical procedures that should be available worldwide [8] and the rapidly developing field of ‘global surgery’ aims to provide improved and equitable care to the world’s population [20]. The World Health Organization (WHO) has recently released a new report on the surgical approaches to the urogenital manifestations of LF [6]. Men with uncomplicated hydrocoeles can be treated at district (first level) hospitals, whereas those with more complicated hydrocoeles may need more experienced surgeons at regional or provincial (second or third level) hospitals [2,6,7,21]. The cost of surgery varies by country, but is estimated to range between US$80 and US$360 [6,22], and considered to be a very cost-effective intervention if less than US$66 [23]

Several studies have shown that men benefit from surgery with improved physical outcomes and relatively low post-operative complications and recurrence rates, however, the results are variable and not systematically reported [7,24–28]. In Ghana, a post-surgery follow-up survey including focus groups discussions and in-depth interviews found that patients reported major improvements in their health, physical and work capacity, social status and sexual performance [26]. In Malawi, a retrospective physical and socio-economic survey on men who had surgery between 2010 and 2012 as part of hydrocoele camps or routine procedures, found that both younger and older men reported significant positive changes across many aspects of their lives, especially with respect to their mobility, pain, mental health and economic situation [28].

Malawi has made good progress towards the elimination of LF as a public health problem, and in recent years has scaled-up MMDP activities, including hydrocoele surgeries to address the national burden. The highest number of hydrocoele cases occur in the highest endemic districts of Chikwawa and Nsanje in the southern region of the country [29,30], The Malawi LF elimination programme conducted its first large scale hydrocoele camp in these two districts in 2015, which provided an opportunity to build on and confirm previous results [28] and prospectively recruit men pre-surgery to better understand the range of clinical conditions and barriers to surgery, and to follow them after surgery to assess the potential impact of surgery on the quality of their lives.

## Methods

### Study area

This study was conducted in Chikwawa and Nsanje Districts located in the Southern Region of Malawi, which had estimated populations of 549,706 (50.2% male) and 288,581 (48.2% male) respectively in 2015 [31]. These districts had the highest baseline LF prevalence at the beginning of the National LF Elimination Programme in 2008 ranging from 15.3% to 79.1% [29,30], and had recorded a high LF morbidity burden in 2015 during mapping activities (described below), including 1867 men with hydrocoele; making these priority districts for MMDP intervention strategies.

### Study design

**Fig 1:**
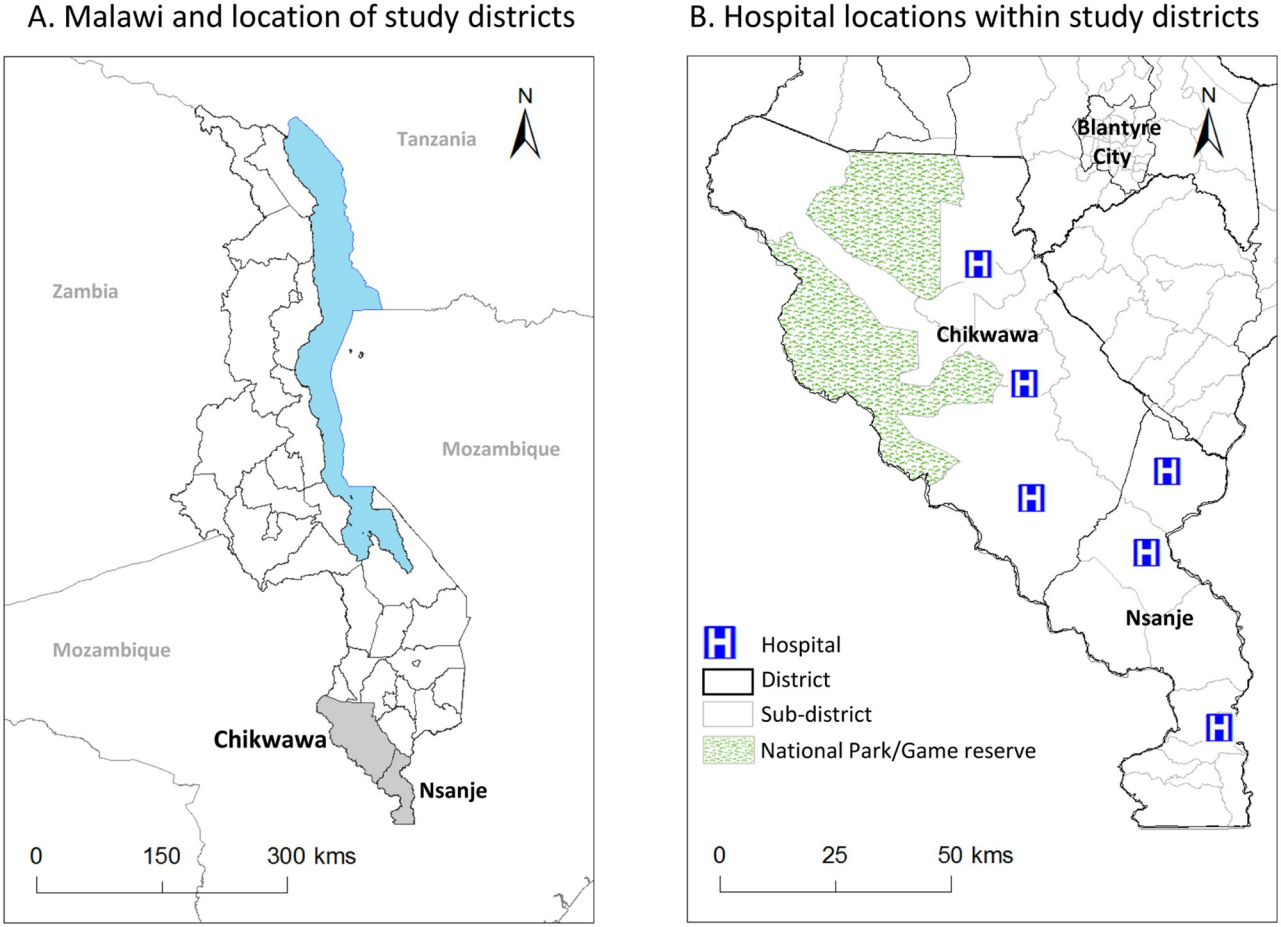
Location of hospitals in study districts of Chikwawa and Nsanje.

We prospectively recruited and followed-up men aged 18 years and older, who were identified in their community and consented to having surgery for their hydrocoele (i.e. hydrocelectomy) as part of a LF Elimination Programme free hydrocoele camp, coordinated by the Ministry of Health (MoH). The hydrocoele camp was planned to be conducted in six hospitals across Chikwawa and Nsanje Districts in December 2015. Each hospital aimed to conduct up to 100 surgeries over a 7 to 10-day period with hospital staff, operating theatres, and post-surgery beds specifically allocated for this large-scale surgical intervention. The study districts and location of the hospitals are shown in Fig 1, which were mapped in ArcGIS 10.5.1 (ESRI, Redlands, CA) using traditional area boundaries available from https://data.humdata.org/dataset/malawi-administrative-level-0-3-boundaries.

Patients were initially identified by local health surveillance assistants (HSAs) during detailed morbidity mapping activities conducted in 2014 and 2015, where every household was visited, and men with hydrocoele registered and reported [32]. The LF Programme team, District Health Officers and HSAs were responsible for raising awareness of the camp and mobilising men for surgery, which was done through their routine health facility communication network. The hydrocoele camp was the first one planned by the MOH to address the needs of the 1867 men identified with hydrocoele across the two districts.

The surgery was conducted by experienced local surgeons or medical officers and supported by nursing staff who routinely conduct hydrocoele surgeries in their hospital or have previously participated in hydrocoele camps in the area [28]. Pre-surgery, surgeons or medical officers were responsible for assessing men and clinically diagnosing their condition as a hydrocoele. The standard hydrocoelectomy procedures were followed and the operations were conducted using the resection (excision technique) surgical technique with a local or spinal anaesthesia, and patients discharged one or two days after surgery with analgesia. Oral antibiotics were prescribed post-surgery for 7–14 days depending on the hospital discharge regimen, including erythromycin, azithromycin or ciprofloxacin and if men returned with heavy infection, they were given intravenous ceftriaxone or metronidazole. Patients were advised to report to their local HSA or back to the hospital if there were any post-operative complications or symptoms of concerns such as swelling, bleeding, infection, pain or bandage issues.

Pre-surgery, information on individual demographic characteristics, clinical condition, barriers to surgery, and quality of life (QoL) was recorded through a semi-structured questionnaire (indicators described below). One nurse (male or female) per hospital was trained and supervised by the field research team to administer the questionnaire to men presenting for surgery. Post-surgery surveys were conducted at 3-months (March 2016) and 6-months (June 2016) by the field research team, and a HSA. Men were randomly selected from the list of pre-surgery patients and stratified across the six hospitals. Men were visited in their homes or at the nearest health facility and the same questionnaire, adapted to account for the absence of a hydrocoele following surgery, with additional information related to their recovery, was administered. All interviews were conducted in private. If men were not available, the reason for loss to follow-up was recorded. Post-surgery, a projected sample size of 155 (taking into account potential attrition) at 6-months post-surgery was designed to detect an effect of 20% for the entire cohort at 95% confidence and at a power of 0.8. More detailed assessment by age group and hydrocoele stage was carried out after sampling was completed with more details provided in the data collection and analysis section below.

### Study indicators

#### Pre-surgery characteristics, conditions and causes

Demographic information included place of residence (district), health catchment (name), length of time living in the district (years), age (years), marriage status (married, widowed, divorced, single), highest level of education (no schooling, primary, secondary, post-secondary/vocational) and primary source of income (paid employment, own business, own vegetable garden/animal, housework, family support, charity, other).

Clinical condition information included the length of time men had had the hydrocoele (years), and four clinical criteria based on classifications described in detail and pictorially presented by Capuano and Capuano [33] including

i) Type of hydrocoele–unilateral or bilateral
ii) Side of hydrocoele–right or left side of the scrotum, or bilateral
iii) Size of the hydrocoele–Stages I to VI; briefly Stage I (scrotum size of tennis ball), Stage II (greater than tennis ball), Stage III (reaches mid-thigh), Stage IV (reaches knee), Stage V (reaches between knee and lower mid-leg) and Stage VI (reaches between lower mid-leg and ankle)
iv) Penis burial—Grades 0 to 4; briefly Grade 0 (no burial), Grade 1 (partial burial with visible part of penis more than 2cm), Grade 2 (partial burial with visible part of penis more than 2cm), Grade 3 (total burial with glans penis visible) and Grade 4 (total burial with glans penis invisible)

Barriers to men having surgery was based on information, which included i) the main reason why they had not had surgery before (didn’t know it was possible, condition wasn’t bad, was scared, didn’t want to leave family, couldn’t afford surgery/transport/accommodation, no transport available, couldn’t afford time off work, other) ii) their knowledge of the disease that causes hydrocoele (LF/elephantiasis, don’t know, other) iii) knowledge of the cause of the disease (mosquitoes, worms, sexually transmitted, bad water, nothing/bad luck, witchcraft, catch off someone, don’t know, other) and iv) the source of information on the cause of the disease (common knowledge, health worker, media–radio/tv, friends, other).

#### Pre- and post-surgery comparison

Recovery from surgery information was based on men’s self-reported post-operative symptoms/complications around the incision site including i) if they experienced swelling, bleeding, infection and/or pain/discomfort and i) what type of treatment they received. The frequency and severity of the post-operative symptoms and detailed information of their treatment were not recorded or verified by a health worker as part of this study, and therefore analysis of these variables, were limited due to the lack of detail, and potential for recall bias and/or subjective bias.

Quality of life (QoL) was based on information from an adapted Lymphatic Filariasis Quality of Life Questionnaire (LFSQQ)[34,35], and included six predefined domains: pain, mobility, usual activities, self-care, social issues, and psychological health. Each QoL domain consisted of four to six questions (27 questions in total) with men’s answers coded in four levels to assess and score the level of problem the hydrocoele (pre-surgery) or groin area (post-surgery) caused to their QoL. The four levels and associated scores included no problem = 0, mild problem = 1, moderate problem = 2, severe problem = 3. The individual scores were used to calculate an overall QoL score, which ranged between 0 (no problems recorded in all 27 questions) and 81 (severe problems recorded in all 27 questions) and a mean QoL score for each domain. In addition, the overall and domain-specific QoL scores were stratified by age group and hydrocoele stage.

Men were asked if the hydrocoele (pre-surgery) or groin area (post-surgery)

- Pain (5 questions): gives them gives them pain or discomfort 1. sitting or lying still 2. generally moving around the house doing every-day activities 3. walking 4. doing strenuous physical work 5. having sex
- Mobility (6 questions): gives them problems 6. moving in and around your home 7. sitting or getting out of a chair 8. lying down or standing up from the floor. 9. going up steps/stairs 10. using public transport 11. walking for one hour at a normal walking pace without a break
- Usual activities (4 questions): gives them problems 12. doing your job 13. doing household activities 14. doing usual leisure activities 15. having sex
- Self-care (4 questions): gives them problems 16. dressing or bathing yourself 17. urinating 18. using the toilet by themselves 19. accessing medical treatment when need it
- Social issues (4 questions): gives them problems 20. moving around freely in public and interacting with people they don’t know well 21. joining in regular social activities outside the home 22. joining in regular social activities inside the home or homes of family and friends 23. with romantic relationships
- Psychological health (4 questions): makes them feel 24. worried about their health 25. worried about the future 26. neglected by friends and family members 27. unable to make plans for the future

Economic information was based on men’s ability to work including i) the estimated number of days (adding partial days together with full days) they had been unable to work in the last month due to their hydrocoele (pre-surgery) or groin area (post-surgery).

### Data collection and analysis

Survey information was collected using OpenDataKit (ODK; https://opendatakit.org) made available on an electronic tablet and transferred to IBM SPSS Statistics 24 for descriptive and statistical analysis. The use of ODK helped to ensure all questions were answered and eliminate the problem of missing data. Individuals were grouped into younger and older groups using the mean age of the pre-surgery sample to account for differences by age. Individuals were also grouped by hydrocele stage Stages I and II = Mild/Moderate; Stages III, IV and V = Severe. Stage III hydrocoeles (reaches mid-thigh) were grouped with Stages IV and V, as they were considered to have a significant impact on men’s QoL. It is acknowledged that this grouping may dilute the potential effect of severity.

Pre-surgery, Chi-square tests were used to examine differences (clinical condition, barriers to surgery and knowledge of the cause of hydrocele) between age groups. Post-surgery at both 3-months and 6 months, Chi-square tests were used to compare reported symptoms/complications from surgery between the age group and hydrocoele stage group.

For the QoL analysis, student’s t test (paired) were used to compare responses from men at different time points. When comparing responses between pre-surgery and post-surgery at 3-months, only individuals who had been interviewed at 3-months post-surgery were included in the analysis. When comparing responses between 6-months post-surgery and earlier time points, only individuals that had been interviewed at 6-months post-surgery were included in the analysis.

### Ethics statement

Ethical approval for this study was obtained from the Research Ethics Committee at the Liverpool School of Tropical Medicine, UK (Research Protocol 15.047), and the National Health Sciences Research Committee in Malawi (Research Protocol 15/3/1406). All adult participants provided written informed consent, no children participated in the study.

### Patient and public involvement statement

This research was conducted without any patient and public involvement.

## Results

### Pre-surgery characteristics, conditions and causes

#### Demographic characteristics

**Fig 2:**
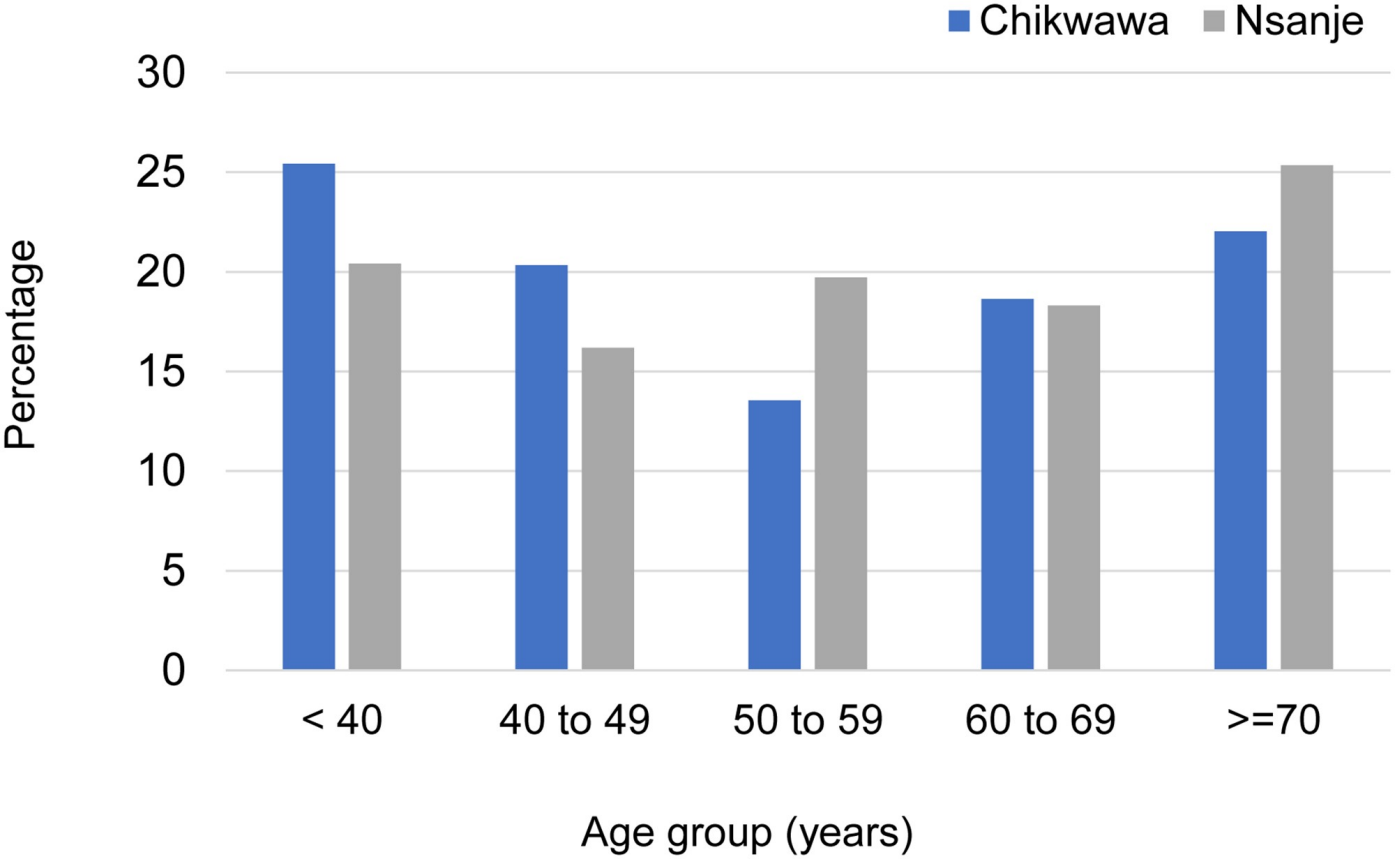
Age distribution of men having surgery for hydrocoele in Chikwawa and Nsanje Districts.

A total of 326 men received surgery across the six hospitals during the hydrocoele camp in November (n = 125) and December (n = 201) 2015. The surgeries started in selected hospitals in late November to fit with their schedules and staff availability, which resulted in these men being excluded from the planned survey that was due to begin in December. In addition, three men reported inguinal hernias and were referred for surgery following the hydrocoele camp. In total, 201 men were interviewed pre-surgery in December 2015; 61 men (30.3%) from three Chikwawa hospitals and 140 men (69.7%) from three Nsanje hospitals. The 201 men were from 31 health catchment areas and had predominately lived in their district all their lives (80.1%) or for more than 10 years (17.4%). Their age ranged from 24 to 99 years (mean 54.9 years SD = 16.6); the age group distribution for each district is shown in Fig 2. There were 95 men younger than the mean aged <55 years, and 106 men older than the mean aged ≥55 years. Most men were married (90%) and had a primary school level of education (64.7%) or no schooling (26.9%). Their primary source of income was their own vegetable garden (49.8%), own business (13.4%), paid employment (10.5%), housework (9.0%), farming or fishing (8.5%) with a small proportion relying on piecework, charity and/or family support.

#### Clinical conditions

Men reported having their hydrocoele for an average of 11.4 years (10.9 years for younger men; 11.8 years for older men), with a range of 1 to 50 years. Pre-surgery hydrocoele conditions, which were assessed by a medical professional before surgery are summarised by age group in Table 1. Most men had unilateral hydrocoeles (65.2%), affecting the left scrotum (36.3%) more than the right (28.9%). Overall, a significant difference was found between the younger and older age groups (X^2^ = 5.978, p = 0.05) with younger men found to have a high proportion of left scrotums affected, and older men had a higher proportion of bilateral hydrocoele. Most men had hydrocoele Stage I (13.9%), Stage II (43.8%) or Stage III (31.3%), and penis burial Grade 0 (44.8%), Grade 1 (39.3%) or Grade 2 (12.9%). Twenty men had hydrocoele Stage IV (10%), 14 men were in the older age group, with five men ≥75 years of age and penis burial Grades 1,2 and 4. Two men had hydrocoele Stage V, aged 75 years with penis burial Grade 3 and aged 48 years with penis burial Grade 4. Overall, no significant differences were found between younger and older men with hydrocoele stage or penis burial (Table 1).

**Table 1:** Clinical classification of patient’s hydrocoele by age group.

Presentation	Total	<55 years	≥55 years	Chi-square
**Side affected**				
Left	73 (36.3%)	42 (44.2%)	31 (29.2%)	X^2^ = 5.978 P = 0.05*
Right	58 (28.9%)	27 (28.4%)	31 (29.2%)	
Bilateral	70 (34.8%)	26 (27.4%)	44 (41.5%)	
**Hydrocoele size**				
Stage I	28 (13.9%)	16 (16.8%)	12 (11.4%)	X^2^ = 2.191 P = 0.534
Stage II	88 (43.8%)	42 (44.2%)	46 (43.4%)	
Stage III	63 (31.3%)	29 (30.5%)	34 (32.1%)	
Stage IV or V	22 (10.9%)	8 (8.4%)	14 (13.2%)	
**Penis burial**				
Grade 0	90 (44.8%)	49 (51.6%)	41 (38.7%)	X^2^ = 3.644 P = 0.303
Grade 1	79 (39.3%)	33 (34.7%)	46 (43.4%)	
Grade 2	26 (12.9%)	10 (10.5%)	16 (15.1%)	
Grade 3 or 4	6 (3.0%)	3 (3.2%)	3 (2.8%)	
**Total**	**201 (100%)**	**95**	**106**	

*p-value significant at 0.05

#### Barriers to surgery

Men reported the main barriers to hydrocoele surgery as not being able to afford surgery, including associated transport and accommodation costs (36.3%), not thinking their condition was bad enough (16.4%), being scared or ashamed (11.9%) or not knowing surgery was possible or available (10%) (Table 2). Reasons related to hospital resources (7.0%), work and/or family commitments (7.5%), tradition or local medicines (5.5%) and medical reasons (5.5%) were also reported as barriers to surgery. There was no significant difference between younger and older men in reporting their barriers to surgery.

**Table 2:** Barriers to hydrocoele surgery reported pre-surgery by age group.

Barriers to surgery	Total No. (%)	<55 years	≥55 years	Chi-Square
Couldn't afford surgery/transport/accommodation	73 (36.3%)	33 (34.7%)	40 (37.7%)	X^2^ = 4.665 P = 0.793
Condition wasn't bad enough	33 (16.4%)	17 (17.9%)	16 (15.1%)	
Scared or ashamed	24 (11.9%)	13 (13.7%)	11 (10.4%)	
Didn't know surgery was possible	20 (10%)	7 (7.4%)	13 (12.3%)	
Resource reasons: Surgery was postponed/not available/lack of resources/no transport	14 (7%)	6 (6.3%)	8 (7.5%)	
Couldn’t take time off work/family commitments	15 (7.5%)	9 (9.5%)	6 (5.7%)	
Was using traditional/local medicines	11 (5.5%)	6 (6.3%)	5 (4.7%)	
Medical reasons: Had surgery previously but hydrocoele had reoccurred/ was not fit for surgery	11 (5.5%)	4 (4.2%)	7 (6.6%)	
**Total**	**201 (100%)**	**95 (100%)**	**106 (100%)**	

Men reported the cause of their hydrocoele as sexually transmitted (22.4%), having intercourse with a woman in menses or soon after abortion or giving birth (16.9%), bad luck (15.9%), or mosquitoes (8.5%). Nearly one fifth of men stated that they did not know what caused their hydrocoele (18.4%) and a few men reported other factors such as bad weather, not heeding tradition, infidelity, poor hygiene, witchcraft and hereditary (Table 3). There was no significant difference between young and older men. Of the men who reported the cause of their hydrocoele (n = 164), nearly half received their information from a friend (45.7%) and one third stated that it was common knowledge (32.3%) (Table 4). Approximately 10% of men received information from community health workers, and of these, the majority (88%) reported mosquitoes as the cause of hydroceles. The other two men that received information from a community health worker reported water and infection as the cause of their hydrocoele (Table 4).

**Table 3:** Reported causes of hydrocoele—Pre-surgery.

Causes of hydrocoele	Total No. (%)	<55 years	≥55 years	Chi-Square
Sexually transmitted	45 (22.4%)	21 (22.1%)	24 (22.6%)	X^2^ = 10.290 P = 0.67
Don't Know	37 (18.4%)	14 (14.7%)	23 (21.7%)	
Sex with a woman on menses, after abortion/ giving birth	34 (16.9%)	22 (23.2%)	12 (11.3%)	
Nothing/Bad luck	32 15.9%)	11 (11.6%)	21 (19.8%)	
Other	23 (11.4%)	9 (9.5%)	14 (13.2%)	
Mosquitoes	17 (8.5%)	12 (12.6%)	5 (4.7%)	
Catch off someone (Infectious)	13 (6.5%)	6 (6.3%)	7 (6.6%)	
**Total**	**201 (100%)**	95 **(100%)**	106 (100%)	

*Includes bad weather, worms, not heeding tradition, infidelity, poor hygiene, witchcraft and hereditary

**Table 4:** Information sources on the causes of hydrocoele reported in Tables 2 and 3 (pre-surgery).

Information source	Total Number (%)	Number identifying mosquitoes as the cause of LF
Friends	75(45.7%)	0 (0%)
Common knowledge	53 (32.3%)	0 (0%)
Community health worker	17(10.4%)	15 (88.2%)
Parents/elders/ Radio/TV/media	14(4.5%)	0 (0%)
Don't know	5 (3.0%)	0 (0%)
**Total**	164*(100.0%)	

* Excluding the 37 men that answered “I don’t know” when asked for the cause of hydrocele (Table 3)

### Pre- and post-surgery quality of life comparisons by age and stage

Post-surgery, a total of 152 men at 3-months and 137 men at 6-months were included in the survey. The loss to follow-up was due to men not being available at the time of interview due to travelling, working away, or no longer living in the district. It was not possible to contact men in advance of the interview as the majority lived in rural villages with limited phone access, and the name of the local community health worker was not known or recorded to assist with the follow-up, which is acknowledged as a limitation.

#### Recovery from surgery

Post-surgery at the 3-months survey, 90 men reported they had experienced some degree of swelling, bleeding, infection and/or pain/discomfort around the incision site in the months immediately following surgery; two men reported all symptoms (swelling, bleeding, infection and pain/discomfort); two men (swelling, infection, pain/discomfort), four men (swelling, pain/discomfort), three men (bleeding, pain/discomfort), five men (infection, pain/discomfort), five men (swelling only) and 69 men (pain/discomfort only). Six men reported they returned to hospital for consultation and/or treatment following bleeding and/or infection and had their surgical site re-examined, re-sutured, cleaned and/or dressed. Nine of the men with infection and one man with bleeding, reported they received further antibiotic treatment. Most men with pain/discomfort reported they took oral pain relief e.g. paracetamol.

Specifically, with respect to the men ≥75 years with Stage IV and those with Stage V: three men ≥75 years with Stage IV were included in the follow-up survey and reported no complications other than pain and took oral pain relief. Of the two men with Stage V, the 75 year-old man with penis burial Grade 4 reported no complications other than pain and took oral pain relief. The 48 year-old man with penis burial Grade 4 reported swelling, bleeding, pain, infection 5 days post-surgery and required antibiotics, pain relief and his wound cleaning in hospital.

Post-surgery at 3-months and 6-months, all men reported that their swelling, bleeding, and/or infections had resolved. At 3-months post-surgery, only three men reported on-going pain/discomfort, which had resolved at 6-months post-surgery. Overall, there was no significant difference between younger and older men, or between men with mild/moderate and severe stages of hydrocoele in the post-surgery recovery (Table 5).

**Table 5:** Self-reported post-operative symptoms and complications, reported during surveys conducted at 3-months post-surgery (n = 152).

Domain	Response	Total	Age			Hydrocoele Stage		
			<55	≥55 years	Chi square	Mild/ Moderate	Severe	Chi square
**Swelling**	No	139	70 (94.5%)	69 (88.5%)	X^2^ = 1.826 P = 0.289	87 (92.6%)	52 (89.7%)	X^2^ = 0.385 P = 0.535
	Yes	13	4 (5.5%)	9 (11.5%)		7 (7.4%)	6 (10.3%)	
**Bleeding**	No	147	71 (96.0%)	76 (97.4%)	X^2^ = 0.265 P = 0.607	93 (98.9%)	54 (93.1%)	X^2^ = 3.836 P = 0.070
	Yes	5	3 (4.0%)	2 (2.6%)		1 (1.1%)	4 (6.9%)	
**Infection**	No	143	69 (93.2%)	74 (94.9%)	X^2^ = 0.181 P = 0.671	89 (94.7%)	54 (93.1%)	X^2^ = 0.689 P = 0.471
	Yes	9	5 (6.8%)	4 (5.1%)		5 (5.3%)	4 (6.9%)	
**Pain / discomfort**	No	77	33 (44.6%)	34 (43.6%)	X^2^ = 0.016 P = 0.901	33 (44.6%)	34 (43.6%)	X^2^ = 0.230 P = 0.151
	Yes	85	41 (55.4%)	44 (56.4%)		41 (55.4%)	44 (56.4%)	
**Total**			**74 (100%)**	**78 (100%)**		**(94 (100%)**	**(58 (100%)**	

*p-value significant at 0.05

Note. Men were asked in the 3-months post-surgery if they experienced swelling, bleeding, infection and/or pain/discomfort following the surgery

#### Patient quality of life

**Fig 3:**
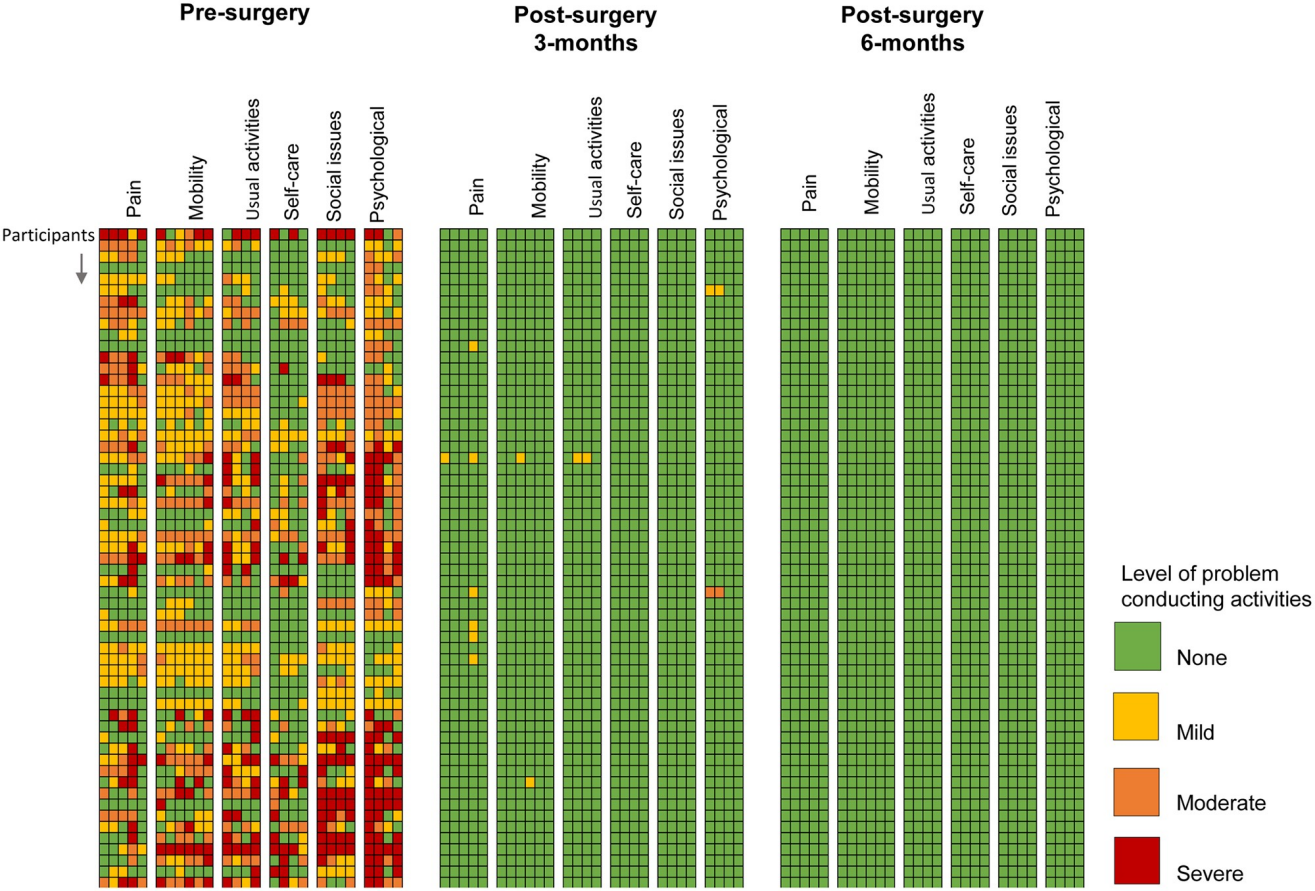
Quality of life responses across six domains colour-coded by the level of problem a subset of men experienced pre-surgery and post-surgery.

Pre-surgery, the overall mean QoL score was 30.4 (SD = 16.1) with mean domain scores ranging from 2.2 to 6.7 (Table 6). In contrast, the overall mean QoL score post-surgery at 3-months was 0.4 (SD = 2.3) with individual mean domain scores ranging from 0 to 0.2, and post-surgery at 6-months were all zero (SD = 0) indicating no problem related to their QoL overall or in any specific domain. Fig 3 shows the colour-coded score for each question in each domain for a subset of men included in the pre-surgery, post-surgery at 3-month and 6-month surveys (n = 137). The colour-coded figure for all men is shown in S1 Fig. The colour-coding of the scores visually highlights the significant changes from pre-surgery problems when many problems were classified as severe (red) and moderate (orange), to the post-surgery problems where most were classified as mild (yellow) and no problem (green).

**Table 6:** Summary of pre-surgery and post-surgery quality of life scores across the domains.

Domain	Pre-surgery Mean score (SD)	3-months follow up mean score (SD)	6-months follow up mean score (SD)	T test results		
				Pre-surgery to 3-months	3 months to 6 months	Pre-surgery to 6 months
**Pain**	5.4 (3.9)	0.2 (0.8)	0 (0)	t = 15.1823, p = 0.000	t = 2.4436, p = 0.016	t = 16.2396 p = 0.000
**Mobility**	6.2 (4.9)	0.1 (0.8)	0 (0)	t = 14.2454, p = 0.000	t = 1.8035, p = 0.074	t = 14.8273 p = 0.000
**Usual activities**	4.7 (3)	0.1 (0.5)	0 (0)	t = 17.815, p = 0.000	t = 1.9339, p = 0.055	t = 18.4616 p = 0.000
**Self-care**	2.2 (2.5)	0 (0.1)	0 (0)	t = 10.4734, p = 0.000	t = 1.7449, p = 0.083	t = 10.5665 p = 0.000
**Social issues**	5.1 (3.9)	0 (0)	0 (0)	t = 15.4897, p = 0.000	N/A	t = 15.4897 p = 0.000
**Psychological health**	6.7 (3.1)	0.1 (0.4)	0 (0)	t = 24.943, p = 0.000	t = 1.6431, p = 0.103	t = 25.6588 p = 0.000
**Overall**	30.4 (16.1)	0.4 (2.3)	0 (0)	t = 21.3902, p = 0.000	t = 2.2763, p = 0.024	t = 22.0892 p = 0.000

Note. Number included was 137, only individual included in the survey at 6-months post-surgery were included in the analysis. The four levels and associated scores included no problem = 0, mild problem = 1, moderate problem = 2, severe problem = 3. The individual scores were used to calculate an overall QoL score, which ranged between 0 (no problems recorded in all 27 questions) and 81 (severe problems recorded in all 27 questions). The mean QoL score for each domain ranged between 0 to 15 for pain (5 questions), and between 0 and 18 for mobility (6 questions) and 0 and 12 for all other domains (4 questions).

*p-value significant at 0.05

The greatest changes in QoL scores and most significant differences were found between pre-surgery and post-surgery at 3-months (Table 7). No significant differences were found between younger and older age groups, but, when QoL scores were stratified by hydrocoele stage (mild/moderate vs. severe), significant differences were found pre-surgery overall and with some individual domains (Table 7). Pre-surgery, men with severe stage hydrocoele reported more problems with their QoL than men with mild/moderate stage hydrocoele overall (mean score 35 vs. 27.3) and related to their mobility (mean score 5.5 vs. 7.2), usual activities (mean score 4.2 vs. 5.5), self-care (mean score 1.7 vs. 3) and social issues (mean score 4.4 vs. 6.1), which all became non-significant post-surgery at 3-months (Table 7).

**Table 7:** Summary of pre-surgery and post-surgery quality of life scores across the domains, stratified by age group and hydrocoele stage.

Domain	Time	Total (SD)	Age Group			Hydrocoele Stage		
			<55 (n = 66)	≥55 (n = 71)	T test	Mild/ Moderate (n = 82)	Severe (n = 55)	T Test
**Pain**	Pre-surgery	5.4 (3.9)	5.3 (4)	5.5 (3.9)	t = -0.280 p = 0.780	5.1 (3.5)	5.9 (4.5)	t = -1.128 p = 0.261
	Post 3-months	0 (0)	0 (0)	0.2 (0.9)	t = -1.247 p = 0.214	0.2 (0.8)	0.1 (0.7)	t = 0.414 p = 0.680
**Mobility**	Pre-surgery	6.2 (4.9)	5.8 (5)	6.5 (4.7)	t = -0.794 p = 0.429	5.5 (4.5)	7.2 (5.3)	t = -2.036 p = 0.044*
	Post 3-months	0 (0)	0.1 (0.6)	0.2 (0.9)	t = -0.610 p = 0.543	0.1 (0.6)	0.1 (0.9)	t = -0.132 p = 0.895
**Usual activities**	Pre-surgery	4.7 (3.0)	4.6 (3)	4.8 (3)	t = -0.351 p = 0.726	4.2 (2.8)	5.5 (3.2)	t = -2.355 p = 0.020*
	Post 3-months	0 (0)	0 (0.4)	0.1 (0.6)	t = -0.808 p = 0.421	0.1 (0.4)	0.1 (0.5)	t = 0.149 p = 0.882
**Self-care**	Pre-surgery	2.2 (2.5)	1.9 (2.3)	2.5 (2.6)	t = -1.487 p = 0.140	1.7 (2)	3 (2.9)	t = -3.137 p = 0.002*
	Post 3-months	0 (0)	0 (0.1)	0 (0.2)	= -0.517 p = 0.606	0 (0.2)	0 (0.1)	t = 0.242 p = 0.809
**Social issues**	Pre-surgery	5.1 (3.9)	4.9 (4)	5.3 (3.8)	t = -0.673 p = 0.502	4.4 (3.7)	6.1 (4)	t = -2.579 p = 0.011*
	Post 3-months	0 (0)	0 (0)	0 (0)	N/A	0 (0)	0 (0)	N/A
**Psychological health**	Pre-surgery	6.7 (3.1)	6.8 (3)	6.7 (3.1)	t = 0.241 p = 0.810	6.4 (2.8)	7.3 (3.3)	t = -1.798 p = 0.074
	Post 3-months	0 (0)	0 (0.2)	0.1 (0.5)	t = -0.761 p = 0.448	0.1 (0.5)	0 (0.3)	t = 0.506 p = 0.614
**Overall**	Pre-surgery	30.4 (16.1)	29.4 (16.2)	31.4 (16.1)	t = -0.715 p = 0.476	27.3 (14.2)	35 (17.8)	t = -2.793 p = 0.006*
	Post 3-months	0 (0)	0.2 (1.7)	0.6 (2.6)	t = -0.980 p = 0.329	0.5 (2)	0.4 (2.6)	t = 0.238 p = 0.812

Note. Number included was 137, only individual included in the survey at 6-months post-surgery were included in the analysis. The four levels and associated scores included no problem = 0, mild problem = 1, moderate problem = 2, severe problem = 3. The individual scores were used to calculate an overall QoL score, which ranged between 0 (no problems recorded in all 27 questions) and 81 (severe problems recorded in all 27 questions). The mean QoL score for each domain ranged between 0 to 15 for pain (5 questions), and between 0 and 18 for mobility (6 questions) and 0 and 12 for all other domains (4 questions).

*p-value significant at 0.05

Post-surgery selected patient quotes include: ‘I can run now without a problem’; ‘I am very happy because now I can walk freely and wear normal trousers. I am even leading the village groups’; ‘I feel better now. I am no longer neglected in the community’; ‘I was unable to go to church before as people would stare at me, but now I can go to church’; ‘No-one laughs at me now, or calls me names’; ‘Before the operation I was feeling very weak but now I am able to work and farm. I am healthy again’.

#### Economic outcome

**Fig 4:**
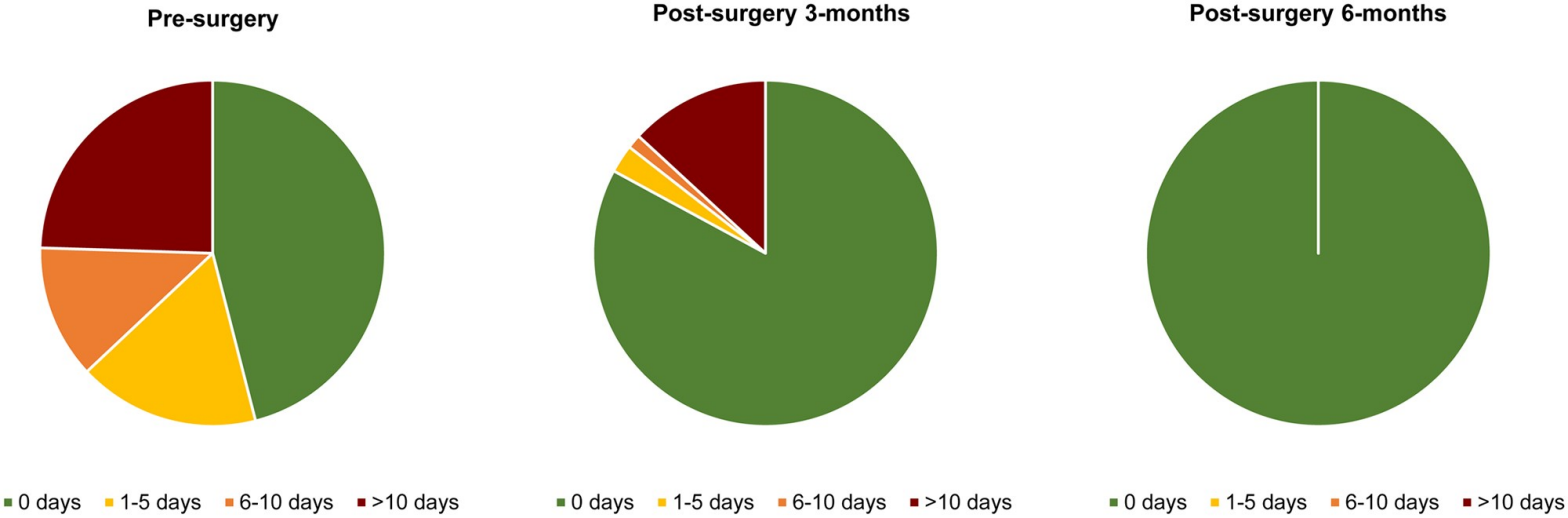
Number of days men have taken off work in the last month at pre-surgery and post-surgery intervals.

Pre-surgery, 54.2% (n = 92/201, 54.2%) of men reported taking at least one day off work (mean = 7.3 (Std. Error 0.75) days off work) in the last month because of their hydrocoele, and of these 24.4% (n = 49/201) reported taking more than 10 days. Post-surgery at 3-months, 17% (n = 26/152) of men reported taking at least one day off work (mean = 3.5 {SE 0.80) days off work) and 13.2% (20/152) more than 10 days off work, and at 6-months this decreased to zero days off work (n = 0/137;mean = 0 days of work) (Fig 4).

## Discussion

This is the first prospective cohort study to quantify the impact of LF hydrocoele surgery on the quality of men’s lives. It builds on the retrospective study by Stanton et al. [28], and better quantifies the clinical burden, knowledge, and barriers to surgery men face before surgery, and the substantial improvements in their physical, social, psychological and economic well-being at 3-months post-surgery, which was maintained and further improved at 6-months post-surgery. The study highlights how local experienced medical teams may be mobilised in Malawi and other endemic countries to address the hidden burden, measure impact and enable national LF elimination programmes to implement the package of care recommended by the GPELF [1]. The hydrocoele camp helped to reduce the number of cases more rapidly than if conducted routinely [28], and mobilised community health workers to engage in programmatic MMDP activities. This is important in high endemic areas such as Chikwawa and Nsanje where around 10% of the hydrocele cases were severe, and one third of cases were bilateral, mainly in older men who may have co-morbidities and require additional care from the health system and their families.

The men presenting for surgery were predominately poor farmers, who had suffered from their condition for around a decade and had their work capacity restricted. Not surprisingly, the main barrier to surgery was the cost, which has been shown to be a problem elsewhere [12,15,26], and for other conditions requiring surgery [20], and emphasises the need for national and international stakeholders to prioritise financial support and access to free surgical services [36], especially for those living far from facilities [28]. Currently, UK Aid provides the largest financial support for hydrocoele surgeries across 25 endemic countries, but more resources are urgently needed to address the immense global burden with quality surgical procedure and potentially improve millions of men’s lives [20,37]. In Malawi, the average cost of the surgery during the hydrocoele camp was US$68 (including surgical supplies, support to surgical and hospital teams, patient transport, food and accommodation), and recent economic analysis of this hydrocoele camp data found a very high benefit-cost ratio of 24.5 [38], with surgery enabling men to lead more economically productive lives. The results were robust to variations in the cost of surgery and the age of departure from the workforce. We found men were able to dramatically increase the number of working days after the initial recovery period, with none of them reporting time off work at 6-months post-surgery.

For men to access and fully benefit from surgery, they need to be better informed of the causes as there were many misconceptions, which is a common problem across endemic countries [10,39]. National LF elimination programmes need to advocate for resources to support social mobilisation to increase community awareness, help to reduce stigma, and increase the uptake and demand for quality surgical services [20]. Building on the existing health system network and optimising health worker programmes will be essential [40], as we found men were more likely to correctly identify the cause of their hydrocoele if the information came from community health workers. Information on their knowledge and participation of mass drug administration was not collected as Malawi was in the surveillance phase but is also an additional consideration. These camps provide an opportunity to provide men with health education about LF and hydrocoele and improve their knowledge, attitude and ability to inform other community members of the benefit of surgery, and should be considered and measured in future studies as changes pre- and post-surgery are likely to be significant. Raising awareness of the presence and impact of inguinal hernias is also important as they may be misdiagnosed as hydrocoeles, but also require surgical care that may transform men’s QoL [8].

Further, support for the cost and access to transport is needed as it is a significant barrier to surgical health care in rural areas of Malawi [41], and for men with hydrocoele as highlighted in this current study, and by Stanton *et al*. [28] who found men living further from the district hospital were less likely to access surgery than those living close. Support for surgeons in these remote regions may also be an option for addressing the burden and the use of new mHealth technologies may help supervise and build capacity as recently shown in Tanzania [42]. These issues for hydocoele as well as groin hernias are important for the global surgery agenda [8,20] and also an integral component of the Sustainable Development Goals (SDGs) Goal 3—Good Health and Well-Being, which aims to end NTDs by 2030 and ensure coverage of interventions and access to essential health services [43].

Overall, the rate of post-surgery complications was low as found in several other countries where they ranged between 2.2–5.0% [7,21,24,27,42]. Most symptoms or complications had resolved at 3-months post-surgery, but a more defined follow-up plan and active involvement of the HSAs, district health workers and surgical teams in the immediate recovery period i.e. first two weeks, may have helped to address and reduce complications further. We recommend that future studies collect more information on the health systems supporting these activities and that patients are followed daily or weekly for one month immediately after surgery to better understand the range, severity and frequency of symptoms and/or complications and whether they differ by age group and/or hydrocoele stage. Further, more systematic reporting from the community health networks may have helped to reduce self-reported bias of post-surgery symptoms and complications, which are subjective and potentially underestimate some of the risks men face. A review of 350 cases in India reported higher complication rates, but noted these had been resolved and surgery led to an improvement in men’s physical mobility, potency and sexual habits and a decrease of mental anguish [25].

Our study also highlights significant improvements in men’s mental health following surgery. This is important as very little is known about mental health illness associated with hydrocoele, or the wider implications for their families and caregivers [4], who may indirectly benefit from interventions [44]. The psychosocial impact and economic consequences of hydrocoeles are potentially huge and quite debilitating as shown with other NTDs [45,46]. These are likely to be more pronounced in men with more severe conditions who indicated pre-surgery they had a poorer QoL, including difficulty with their mobility. More systematic methods of measuring the burden, comparing the severity and how surgical interventions impact on men’s lives need to be considered, together with ways to prioritise essential surgical services, and strengthen and integrate care into health systems in endemic countries [8,20].

The use of DALYs more systematically together with quality-adjusted life years (QALYs) may provide a better picture of the burden in men and how that may change with surgery. However, it is noteworthy that there are controversies with these health burden measures [47,48], which need to be considered carefully for a disease such as LF where the burden may differ by clinical condition, severity of condition, presence of an ADLA and/or depressive illness [4,5]. It would also be important to understand the range of co-morbidities that may impact on these measures, which has not been considered, but may be particularly prevalent in older men.

While this study highlights that surgery had a significant positive and lasting impact on men’s lives, there were several limitations that should be considered in this study and addressed in future surveys, including i) the lack of detailed pre-surgery clinical data on hydrocoeles, hernias and other pre-existing medical conditions that may impact on patient’s recovery and influence the results ii) the reliance on patient recall of post-surgery symptoms and complications rather than clinical data systematically collected by a trained health worker iii) the risk over interpreting self-reported changes in the QoL as patients may have felt obliged to over-emphasise their positive outcome in the presence of the interviewer iv) the loss to follow-up of many patients and lack of information on reasons why individuals could not be located v) the inability to address clinical follow-up needs of patients with severe stage hydrocoeles and/or complex repairs and vi) the exclusion of additional mental health objective measures to better understand the impact on patients.

### Conclusions

This study prospectively examined men who underwent hydrocoele surgery and highlights the pre-surgical clinical burden, misconceptions about the cause, barriers to surgery and how the surgical intervention significantly improved many aspects of their lives including pain, mobility, daily living activity, social interactions and mental health. Further, it highlights the feasibility of using local medical infrastructure and expertise to address a neglected disease and support national programmes to implement and integrate an essential package of care into the health system. Finally, the survey methods used in this study provide a template to use and develop further by other researchers and programme managers to help assess the impact of interventions and provide evidence of improved QoL of people affected by LF and other NTDs.

## Supporting information

S1 STROBE Checklist (DOC)

S1 Fig Quality of life responses across six domains colour-coded by the level of problem that all men experienced pre-surgery and post-surgery. (TIF)

## References

[1] World Health Organization. Lymphatic Filariasis [Internet]. 2019. https://www.who.int/lymphatic_filariasis/en/

[2] World Health Organization. Lymphatic filariasis: Managing morbidity and preventing disability. [Internet]. Geneva; 2013. http://www.who.int/lymphatic_filariasis/resources/9789241505291/en/

[3] Collaborators G 2017 D and II and P. Global, regional, and national incidence, prevalence, and years lived with disability for 354 diseases and injuries for 195 countries and territories, 1990–2017: a systematic analysis for the Global Burden of Disease Study 2017. Lancet. 2018;392: 1789–1858. 10.1016/S0140-6736(18)32279-7 30496104 PMC6227754

[4] Ton TG, Mackenzie C, Molyneux D. The burden of mental health in lymphatic filariasis. Infect Dis Poverty.2015;4: 34 10.1186/s40249-015-0068-7 26229599 PMC4520254

[5] Mathew CG, Bettis AA, Chu BK, English M, Ottesen EA, Bradley MH, et al The health and economic burden of lymphatic filariasis prior to mass drug administration programmes. Clin Infect Dis.2019; 10.1093/cid/ciz671 PMC7286370 31343064

[6] World Health Organization. Surgical approaches to the urogenital manifestations of lymphatic filariasis. Report from an informal consultation among experts. (WHO/CDS/NTD/PCT/2019.04); 2019.

[7] Lim KHA, Speare R, Thomas G, Graves P. Surgical Treatment of Genital Manifestations of Lymphatic Filariasis: A Systematic Review. World J Surg. 2015;39: 2885–2899. 10.1007/s00268-015-3220-4 26319260

[8] Beard J, Ohese-Yeboah M, DeVries C, Schecter W. Hernia and hydrocoele In: Debas H, Donkor P, Gawande A, Jamison D, Kruk M, Mock C, editors. In: Disease Control Priorities (third edition): Volume 1, Essential surgery. Third. 2015 http://dcp-3.org/surgery 26740991

[9] Otabil KB, Tenkorang SB. Filarial hydrocele: A neglected condition of a neglected tropical disease. J Infect Dev Ctries. 2015;9: 456–462. 10.3855/jidc.5346 25989164

[10] Addiss DG, Brady MA. Morbidity management in the Global Programme to Eliminate Lymphatic Filariasis: a review of the scientific literature. Filaria J. 2007;6: 2 10.1186/1475-2883-6-2 17302976 PMC1828725

[11] Zeldenryk LM, Gray M, Speare R, Gordon S, Melrose W. The emerging story of disability associated with lymphatic filariasis: A critical review. PLoS Negl Trop Dis. 2011;5: e1366 10.1371/journal.pntd.0001366 22216361 PMC3246437

[12] Gyapong JO, Gyapong M, Evans DB, Aikins MK, Adjei S. The economic burden of lymphatic filariasis in northern Ghana. Ann Trop Med Parasitol.1996;90: 39–48. 10.1080/00034983.1996.11813024 8729626

[13] Dreyer G, Norões J, Addiss D. The silent burden of sexual disability associated with lymphatic filariasis. Acta Trop. 1997;63: 57–60. 10.1016/s0001-706x(96)00604-3 9083585

[14] Ahorlu CK, Dunyo SK, Koram KA, Nkrumah FK, Aagaard-Hansen J, Simonsen PE. Lymphatic filariasis related perceptions and practices on the coast of Ghana: implications for prevention and control. Acta Trop. 1999;73: 251–261. 10.1016/s0001-706x(99)00037-6 10546843

[15] Gyapong M, Gyapong J, Weiss M, Tanner M. The burden of hydrocele on men in Northern Ghana. Acta Trop. 2000;77: 287–294. 10.1016/s0001-706x(00)00145-5 11114391

[16] Ramaiah KD, Das PK, Michael E, Guyatt HL. The economic burden of lymphatic filariasis in India. Parasitol Today. 2000;16: 251–253. 10.1016/s0169-4758(00)01643-4 10827432

[17] Perera M, Whitehead M, Molyneux D, Weerasooriya M, Gunatilleke G. Neglected patients with a neglected disease? A qualitative study of lymphatic filariasis. PLoS Negl Trop Dis. 2007;1: e128 10.1371/journal.pntd.0000128 18060080 PMC2100378

[18] Babu B V., Mishra S, Nayak AN. Marriage, sex, and hydrocele: An ethnographic study on the effect of filarial hydrocele on conjugal life and marriageability from Orissa, India. PLoS Negl Trop Dis. 2009;3: e414 10.1371/journal.pntd.0000414 19381283 PMC2666802

[19] Litt E, Baker MC, Molyneux D. Neglected tropical diseases and mental health: A perspective on comorbidity. Trends in Parasitology. 2012;28: 195–201. 10.1016/j.pt.2012.03.001 22475459

[20] Bath M, Bashford T, Fitzgerald JE. What is ‘global surgery’? Defining the multidisciplinary interface between surgery, anaesthesia and public health. BMJ Glob Heal. 2019;4: e001808. 10.1136/bmjgh-2019-001808 PMC6830053 31749997

[21] Mante SD, Gueye SM. Capacity building for the modified filarial hydrocelectomy technique in West Africa. Acta Trop. Elsevier B.V.; 2011;120: 76–80. 10.1016/j.actatropica.2010.09.008 20920453

[22] World Health Organization/Department of control of neglected tropical diseases. Investing to overcome the global impact of neglected tropical diseases. Third WHO report on neglected tropical diseases [Internet]. https://www.who.int/neglected_diseases/9789241564861/en/

[23] Turner HC, Bettis AA, Chu BK, McFarland DA, Hooper PJ, Mante SD, et al Investment Success in Public Health: An Analysis of the Cost-Effectiveness and Cost-Benefit of the Global Programme to Eliminate Lymphatic Filariasis. Clin Infect Dis. 2017;64: 728–735. 10.1093/cid/ciw835 27956460 PMC5404931

[24] Dandapat MC, Mohapatro SK, Patro SK. Surgery for large hydrocele. Am J Surg. United States; 1984;147: 387–389. 10.1016/0002-9610(84)90173-9 6703213

[25] Dandapat MC, Mohapatro SK, Patro SK. Elephantiasis of the penis and scrotum. A review of 350 cases. Am J Surg. United States; 1985;149: 686–690. 10.1016/s0002-9610(85)80156-2 3993854

[26] Ahorlu CK, Dunyo SK, Asamoah G, Simonsen PE. Consequences of hydrocele and the benefits of hydrocelectomy: a qualitative study in lymphatic filariasis endemic communities on the coast of Ghana. Acta Trop. 2001;80: 215–21. 10.1016/s0001-706x(01)00159-0 11700178

[27] Thomas G, Richards F, Eigege A, Dakum N, Azzuwut M, Sarki J, et al A pilot program of mass surgery weeks for treatment of hydrocele due to lymphatic filariasis in central Nigeria. Am J Trop Med Hyg. 2009;80: 447–451. 19270297

[28] Stanton MC, Smith EL, Martindale S, Mkwanda SZ, Kelly-Hope L a. Exploring hydrocoele surgery accessibility and impact in a lymphatic filariasis endemic area of southern Malawi. Trans R Soc Trop Med Hyg. 2015; 1–10. 25673628 10.1093/trstmh/trv009

[29] Ngwira BMM, Jabu CH, Kanyongoloka H, Mponda M, Crampin AC, Branson K, et al Lymphatic filariasis in the Karonga district of northern Malawi: a prevalence survey. Ann Trop Med Parasitol. 2002;96: 137–144. 10.1179/0003498302125000411 12080974

[30] Ngwira BM, Tambala P, Perez a M, Bowie C, Molyneux DH. The geographical distribution of lymphatic filariasis infection in Malawi. Filaria J. 2007;6: 12 10.1186/1475-2883-6-12 18047646 PMC2233609

[31] The National Statistics Office of Malawi. National Statistics Office [Internet]. http://www.nsomalawi.mw/

[32] Chiphwanya J, Martindale S, Mkwanda S, Matipula D, Ndhlovu P, Mackenzie C, et al. Developing the first national database and map of lymphatic filariasis clinical cases in Malawi. Am Soc Trop Med 66th Meet Balitimore, USA. 2017.

[33] Capuano GP, Capuano C. Surgical management of morbidity due to lymphatic filariasis: the usefulness of a standardized international clinical classification of hydroceles. Trop Biomed. 2012;29: 24–38. 22543600

[34] Aggithaya MG, Narahari SR, Vayalil S, Shefuvan M, Jacob NK, Sushma KV. Self care integrative treatment demonstrated in rural community setting improves health related quality of life of lymphatic filariasis patients in endemic villages. Acta Trop. 2013;126: 198–204. 10.1016/j.actatropica.2013.02.022 23499714

[35] Stanton MC, Yamauchi M, Mkwanda SZ, Ndhlovu P, Matipula DE, Mackenzie C, et al Measuring the physical and economic impact of filarial lymphoedema in Chikwawa district, Malawi: a case-control study. Infect Dis Poverty. 2017;6: 28 10.1186/s40249-017-0241-2 28366168 PMC5376674

[36] Ozgediz D, Riviello R. The “Other” Neglected Diseases in Global Public Health: Surgical Conditions in Sub- Saharan Africa. PloS Med. 2008;5: e121 10.1371/journal.pmed.0050121 18532875 PMC2408612

[37] Department for International Development. Accelerating the Control and Elimination of Neglected Tropical Diseases (ASCEND) [Internet]. 2019. https://supplierportal.dfid.gov.uk/selfservice/pages/public/supplier/publicbulletin/viewPublicNotice.cmd?bm90aWNlSWQ9NzI4MDA%3D

[38] Sawers L, Stillwaggon E, Chiphwanya J, Mkwanda SZ, Betts H, Martindale S, et al Economic benefits and costs of surgery for filarial hydrocele in Malawi. PLoS Negl Trop Dis. 2020;14: e0008003 10.1371/journal.pntd.0008003 32210436 PMC7094819

[39] Evans DB, Gelband H, Vlassoff C. Social and economic factors and the control of lymphatic filariasis: a review. Acta Trop. 1993;53: 1–26. 10.1016/0001-706x(93)90002-s 8096106

[40] World Health Organization. Global strategy on human resources for health: Workforce 2030. Geneva; 2016.

[41] Varela C, Young S, Mkandawire N, Groen RS, Banza L, Viste A. Transportation barriers to access health care for surgical conditionsin Malawi a cross sectional nationwide household survey. BMC Public Health. England; 2019;19: 264. 10.1186/s12889-019-6577-8 PMC6402149 30836995

[42] Akoko L, Mwanga A, Chikawe M, Lutainulwa E, Ngoma D, Nshalla A, et al Supervision and support in surgical practice using mobile platform: a case of mass hydrocele surgeries in remote regions. mHealth. 2019;5: 41 10.21037/mhealth.2019.09.05 31620468 PMC6789301

[43] United Nations. Global indicator framework for the Sustainable Development Goals and targets of the 2030 Agenda for Sustainable Development [Internet]. 2015. https://unstats.un.org/sdgs/indicators/Global%20Indicator%20Framework%20after%20refinement_Eng.pdf

[44] Martindale S, Chiphwanya J, Matipula D, Ndhlovu P, Betts H, Kelly-Hope L.. Hydrocoele surgery for lymphatic filariasis: measuring the impact on patient caregivers in Malawi. Am Soc Trop Med 66th Meet Balitimore, USA. 2017.

[45] Bailey F, Eaton J, Jidda M, Van Brakel WH, Addiss DG, Molyneux DH. Neglected Tropical Diseases and Mental Health: Progress, Partnerships, and Integration. Trends Parasitol. 2018;35: 23–31. 10.1016/j.pt.2018.11.001 30578149

[46] Hamill LC, Haslam D, Hill B, Dixon R, Burgess H, Jensen K, et al People are neglected, not diseases: the relationship between disability and neglected tropical diseases. Trans R Soc Trop Med Hyg. 2019; 113: 1–6. 31111941 10.1093/trstmh/trz036 PMC6903785

[47] Guyatt HL, Evans D. Economic Considerations for Helminth Control. Parasitol Today. 1992;8: 397–402. 10.1016/0169-4758(92)90187-7 15463552

[48] Payne RJH, Turner L, Morgan ER. Inappropriate measures of population health for parasitic disease? Trends Parasitol. 2009;25: 393–395. 10.1016/j.pt.2009.05.013 19720565

