# Supplementary figures and images for: Significant improvement in quality of life following surgery for hydrocoele caused by lymphatic filariasis in Malawi: A prospective cohort study
Source: PLoS Negl Trop Dis. 2020 May 8;14(5):e0008314. doi: 10.1371/journal.pntd.0008314 (PMC7239494; doi:10.1371/journal.pntd.0008314)

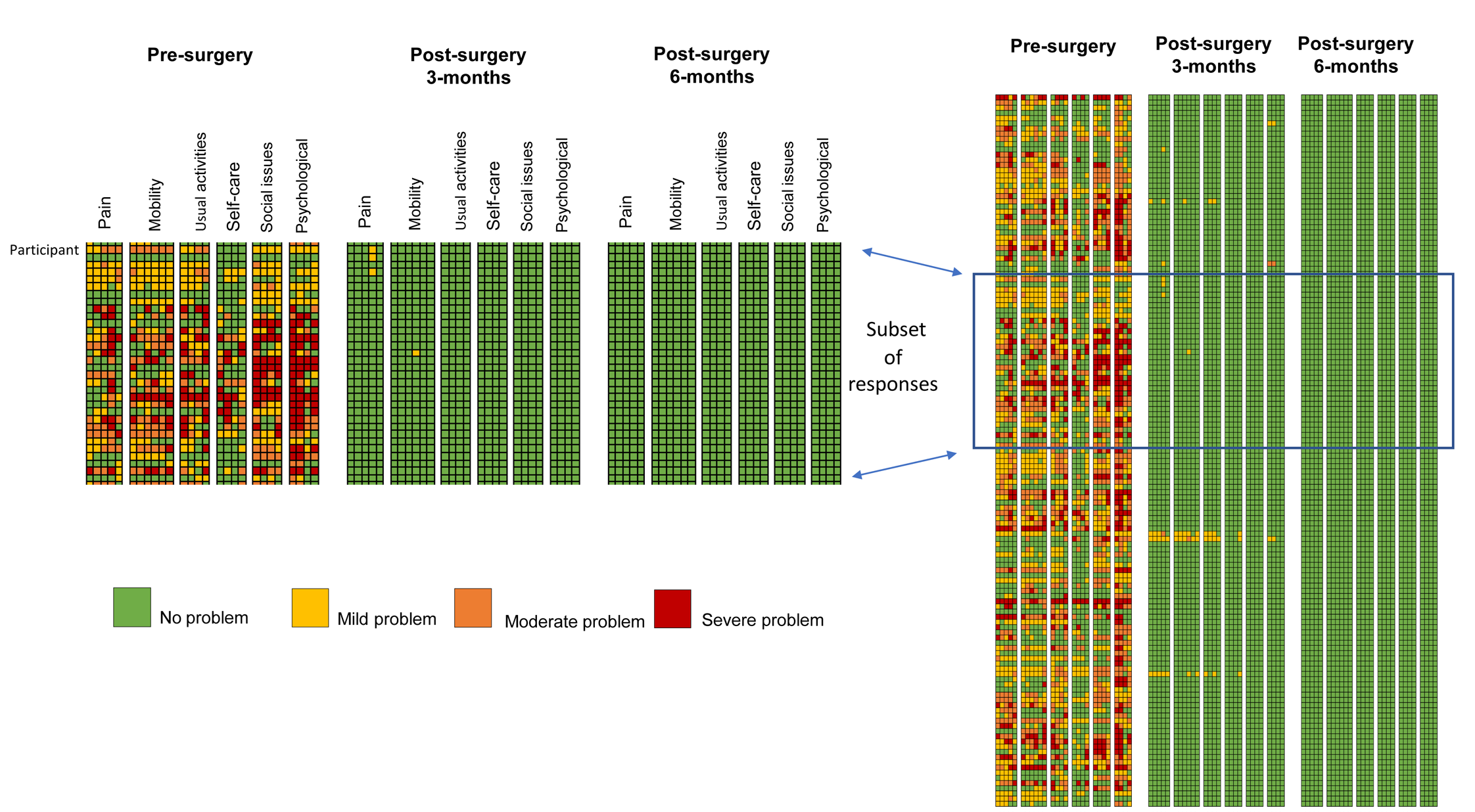

Supplement: S1 Fig — (TIF) [file pntd.0008314.s002.tif]
